# American Medical Association.—The Jubilee Meeting

**Published:** 1897-07

**Authors:** 


**A Monthly Review of Medicine and Surgery.**

*EDITORS:*

THOMAS LOTHROP, M. D. - - WM. WARREN POTTER, M. D.

All communications, whether of a literary or business nature, should be addressed to the managing editor: 284 FRANKLIN STREET, BUFFALO, N. Y.

VOL. XXXVI. JULY, 1897. No. 12.

### AMERICAN MEDICAL ASSOCIATION.—THE JUBILEE MEETING.

THE fiftieth anniversary of the founding of the American Medical Association was appropriately celebrated at Philadelphia, June 1–4, 1897. This occasion, in many respects remarkable, is furnishing an opportunity for much comment on the part of medical journals and in most instances they have bestowed unstinted praise upon the management and character of the meeting.

As a matter of fact there is much to commend and somewhat to reprehend in relation to both. The committee of arrangements, under the chairmanship of Dr. Hobart Amory Hare, performed its part of the work in a praiseworthy manner. Dr. Hare, himself, displayed great generalship in the difficult task to which he was assigned and his lieutenants were well chosen. The difficult problem of registration when wellnigh unto 2,000 men must be recorded was as well handled as it can be under a system that requires each committee of arrangements to formulate its own plans.

The interest in this association is always of a two-fold nature,—namely, scientific and social. The scientific work done at Philadelphia will soon be published in the medical journals, hence need not here be commented on at length. The general impression at the meeting was that the scientific part of the program was about up to the usual average of the association—not better, not worse. The president, Dr. Senn, though a great surgeon, made a poor presiding officer. His voice was pitched in a low key, which made it difficult to hear even what he did say. His presidential address, too, was mediocre in character. The addresses of Drs. Flint, Keen and Hamilton were listened to with interest and each made some telling points—Flint in his introductory remarks, Keen in regard to the antivivisectionists, while Hamilton showed himself a master of his whole subject.

The addresses of Mayor Warwick and the Hon. Charles Emory Smith on the first day, and those of the President of the United States and Governor Hastings on the second day, were all neat bits of rhetoric and some of them rose to a high plane of oratory, notably that of Governor Hastings. President McKinley condensed a great speech into a few words—a gift that is natural to him and which few possess.

The jubilee exercises proper on the third day might have been worse and there was abundant room to have made them better. The occasion afforded ample opportunity for hero worshippers to display their enthusiasm and otherwise it was a restful break in the routine. Dr. N. S. Davis read his address in good voice and the subject-matter was an interesting bit of history. The opportunity was here afforded to invite the president of the Medical Society of the State of New York to participate in the proceedings at least by his personal presence; but it was lost, more's the pity. To completely ignore a great medical society, that was founded in 1806 and was the first organised body to give direction to medical education in the United States, in which also the American Medical Association had its birth and after which its form of government was modeled, was in our view an error if not a misfortune. These jubilee exercises were distinct and apart from the ordinary work of the association, and during their conduct it would have been a graceful act for Dr. Davis to have welcomed the president of that society, nor would it have diminished the renown that is so justly accorded to the "Father of the Association" had he displayed a magnanimity that was worthy his exalted position and the occasion. The address of Dr. George Ben Johnston[1], of Richmond, on behalf of the state medical societies is a paper that will bear careful reading, and it furnished a bright coloring to a dark foreground. It was masterful in conception, in length and in material. The National Confederation of State Medical Examining Boards as an organisation was also ignored at these exercises, although the individual boards were invited to participate. Unfortunately, however, they were not heard from on the stage, as the speaker selected by the jubilee committee departed for his home the previous evening.

The social entertainments were of the highest order. It would be difficult to bestow unmerited praise on this part of the program,

1. Published in full on page 933.

Whether in the way of public receptions, luncheons, lawn and theatre parties, or private dinners and entertainments, the whole series was managed with rare discretion. It was no ordinary task to adequately provide for such a number, and Dr. Hare with his lieutenant Dr. de Schweinitz, chairman of the sub-committee on entertainment, conducted these functions in a manner beyond criticism.

The hospitalities of the clubs—the Art, the Union League, the United Service, and others,—were of an exceptional order. The entree was given by card to many of the visitors where luncheons, dinners and many home comforts were enjoyed. There were numerous private luncheons and dinners given by Philadelphians that were of the most enjoyable nature, and these after all furnished the best opportunity for agreeable acquaintanceship with the charming men and women who people the Quaker City. It was hard to keep pace with the rapidity of movement incident to all this round of pleasure, and before the end of a week many of the visitors found it convenient to seek the quietude of Greater New York for rest and recuperation.

We must not omit to mention the many clinics that were thrown open to invited guests where operations of all sorts were witnessed under the most extreme conditions of sanitation and asepsis. The new operating amphitheatre of the Medico-Chirurgical college and hospital afforded an object lesson in showing what money can do when lavishly expended in perfecting and developing surgical asepsis in operative environment. The new clinic room of the old Pennsylvania hospital is a model of aseptic neatness, convenience and perfection, that attracted many visitors who gave unstinted praise to the old institution.

Finally, the excursion to Atlantic City on Friday was participated in by nearly 1,000 excursionists, who were entertained without expense until Saturday night. This afforded an opportunity for those who desired to visit the ocean and even take a dip in the waves, to do so under the pleasantest auspices, and was a generous ending to a week of lavish hospitality. Philadelphia's fame as a host did not suffer at the hands of the committee of arrangements of the jubilee meeting of the American Medical Association.

### Notes and Echoes.

The usual number of amendments to the constitution and by-laws were offered and as usual they were all voted down. It would appear that this association does not care to have any new fashions introduced into its government. The old-time way is good enough for it.

Dr. John H. Musser, of Philadelphia, was appointed to deliver the address on Medicine next year; Dr. John B. Murphy, of Chicago, the address on Surgery, and Dr. S. C. Busey, of Washington, that on State Medicine. It is needless to add that these are all excellent selections.

The Association of American Medical Colleges held two or three sessions under the presidency of Dr. J. M. Bodine, of Louisville. We are not advised as to the nature of its transactions, but it showed great discretion in electing Dr. J. W. Holland, dean of Jefferson, as its president for the ensuing year.

The president, Dr. Senn, appointed Dr. Henry D. Holton, of Brattleboro, Vt., as official delegate to the British Medical Association at Montreal, August 31–September 3, 1897. No more appropriate selection could have been made. Dr. Holton, though one of the older members is one of the more liberal and progressive ones, and will represent the association in its truest sense.

Commodore Albert L. Gihon, Medical Director U. S. Navy (retired) Chairman of the Rush Monument Committee, received substantial encouragement that his labors, so zealously and almost thanklessly given for so many years, are at last to be crowned with success. At least such will be the case if the contributions promised on the first day by the several states should materialise.

The American Medical Editors Association held its annual meeting and banquet at the Hotel Aldine, Tuesday evening, June 1st. While the meeting was an enjoyable one, rendered especially so by the efforts of the president and Dr. Daland, chairman of committee of arrangements, yet experience shows that Monday evening before the meeting of the great association would be a better date for the editors, and it was so agreed for the future. Dr. Hare was reëlected president—a fit action.

The election resulted in the choice of the following-named officers: President, Brigadier-General George M. Sternberg, M. D., Surgeon-General U. S. Army; vice-presidents, Drs. Joseph M. Mathews, Kentucky, J. L. Thompson, Indiana, F. H. Wiggin, New York, and T. J. Happel, Tennessee; treasurer, Dr. H. P. Newman, Illinois, and secretary, Dr. Wm. B. Atkinson, Pennsylvania. The selection of president is an admirable one. Dr. Sternberg by education, training, position and scientific attainments is every way worthy of the succession. Dr. Mathews, first vice-president, is also a man of conspicuous prominence and every way worthy of his office After a spirited contest Denver was selected as the next place of meeting.

The American Academy of Medicine held a largely attended and most interesting meeting May 29th and 31st, at the Continental Hotel, Philadelphia. Its work in creating a public sentiment in favor of higher and better medical education is well known and deserves the highest commendation. Considerable progress was made in the direction named during this meeting. Dr. L. Duncan Bulkley, of New York, was elected president for the ensuing year. Dr. Bulkley was vice-president of the Medical Society of the State of New York, 1896-97, and is now also secretary of the executive committee of the American Medical Association.

The financial budget of the association presented an incongruous monetary problem. The balance at last statement was $9,075.74. The balance now is $5,465.56, showing a loss for the year of $3,610.18. Another curious fact is that the salary of the secretary is $300.00 while the rent paid for the treasurer's office is also $300.00. By a new rule the treasurer is to supervise the registration for which he is to be paid $100.00. The trustees are paid for attendance upon meetings of the board all the way from $119.50, the highest, down to $42.50 the lowest. A new executive committee is created that will undoubtedly present bills for attendance upon the meeting next year.

Denver, which secures the meeting next year, will find it difficult to follow Philadelphia in entertaining the association, and a year from now may be a wiser if not a better city. The *Western Medical Review* relates an amusing incident in the committee of nomination relating to the selection of the place of meeting, as follows:

The fight for place of holding the meeting next year was a lively one while it lasted. While several cities extended an invitation for the association to meet with them, only two went into the fight in earnest—namely, Denver and Columbus, Ohio. The former had an able champion in Dr. J. W. Graham, and the forces of Columbus were led by Dr. C. A. L. Reed. When the vote came to be counted it was seen that the quiet but hard work of Dr. Graham had secured the plum in the ratio of two to one. Columbus had a map, beautifully decorated with red lines running between Columbus and the important cities of the east, to show that Columbus was nearer the center of population. But the good effect that might have resulted from this lesson in geography was demolished when a representative from the Denver delegation got up and quietly said: "Denver does not need a map to let people know where it is situated." The laugh and cheering that followed showed that the point was well received. General satisfaction was evident among the members of the association, as they expect an enjoyable time in Denver. It is to be hoped that the physicians of the west will aid the profession of Denver and Colorado in making the meeting next year a successful one, in point of numbers at least. Denver will attend to making it successful otherwise.

